# Seroprevalence of Antibodies against Chikungunya, Dengue, and Rift Valley Fever Viruses after Febrile Illness Outbreak, Madagascar

**DOI:** 10.3201/eid1811.111036

**Published:** 2012-11

**Authors:** Norbert G. Schwarz, Mirko Girmann, Njary Randriamampionona, Alexandra Bialonski, Deborah Maus, Anne Caroline Krefis, Christine Njarasoa, Jeanne Fleury Rajanalison, Herly Daniel Ramandrisoa, Maurice Lucien Randriarison, Jürgen May, Jonas Schmidt-Chanasit, Raphael Rakotozandrindrainy

**Affiliations:** Bernhard Nocht Institute for Tropical Medicine, Hamburg, Germany (N.G. Schwarz, M. Girmann, A.C. Krefis, J. May);; World Health Organization Collaborating Centre for Arbovirus and Haemorrhagic Fever Reference and Research, Hamburg (A. Bialonski, D. Maus, J. Schmidt-Chanasit);; Université d’Antananarivo, Antananarivo, Madagascar (N. Randriamampionona, R. Rakotozandrindrainy);; Hôpital d’Ambositra, Ambositra, Madagascar (C. Jnarasoa);; Medecin Inspecteur de Tsiroanomandidy, Tsiroanomandidy, Madagascar (J.F. Rajanalison);; Medecin Inspecteur de Moramanga, Moramanga, Madagascar (H.D. Ramandrisoa);; and Centre de Santé de Base Urban de Mananjary,Mananjary, Madagascar (M.L. Randriarison)

**Keywords:** chikungunya virus, dengue virus, Rift Valley fever virus, Madagascar, arboviruses, viruses

## Abstract

In October 2009, two–3 months after an outbreak of a febrile disease with joint pain on the eastern coast of Madagascar, we assessed serologic markers for chikungunya virus (CHIKV), dengue virus (DENV), and Rift Valley fever virus (RVFV) in 1,244 pregnant women at 6 locations. In 2 eastern coast towns, IgG seroprevalence against CHIKV was 45% and 23%; IgM seroprevalence was 28% and 5%. IgG seroprevalence against DENV was 17% and 11%. No anti-DENV IgM was detected. At 4 locations, 450–1,300 m high, IgG seroprevalence against CHIKV was 0%–3%, suggesting CHIKV had not spread to higher inland-altitudes. Four women had IgG against RVFV, probably antibodies from a 2008 epidemic. Most (78%) women from coastal locations with CHIKV-specific IgG reported joint pain and stiffness; 21% reported no symptoms. CHIKV infection was significantly associated with high bodyweight. The outbreak was an isolated CHIKV epidemic without relevant DENV co-transmission.

In October 2009, the sentinel surveillance system for early outbreak detection in Madagascar (*1*) reported an increase of cases of fever with joint pain on the eastern coast. At the beginning of February 2010, chikungunya virus (CHIKV) infection was diagnosed in a patient from the Mananjary district. The International Federation of Red Cross and Red Crescent Societies reported 702 clinically diagnosed cases of chikungunya during February 9–February 15, 2010. Six hundred occurred in the coastal city of Mananjary, and 96 occurred in the small village of Irondro at the crossroads between the towns of Mananjary, Manakara, and Ifanadiana, indicating that the area was a focal point of the epidemic (Figure 1) (*2*).

**Figure 1:**
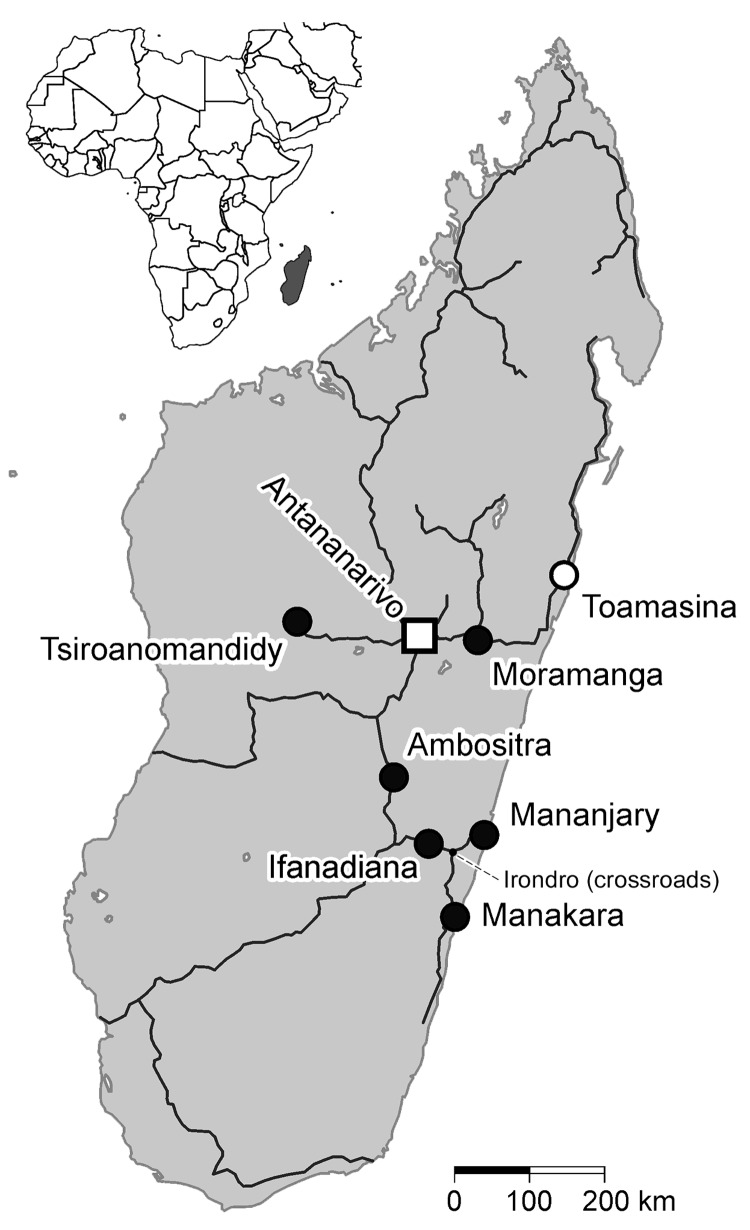
Madagascar (gray shading in inset), showing the main roads, the capital of Antananarivo (square), the harbor city of Toamasina (white circle), and the locations of the 6 study sites (black circles) from which serologic samples from pregnant women were screened for IgG against chikungunya virus, dengue virus, and Rift Valley fever virus. The altitudes of the locations are as follows: Mananjary and Manakar, coastal; Ifanadiana, 466 m; Moramanga, 920 m; Tsiroanomandidy, 860 m; Ambositra, 1,280 m; Toamasina, coastal; Irondro, 40 m; Antananarivo, 1,300 m.

Arthropod-borne viruses (arboviruses) such as CHIKV (*3*), dengue virus (DENV), and Rift Valley fever virus (RVFV) (*4*,*5*) are emerging pathogens in the southwestern Indian Ocean region. In 2005–2006, CHIKV caused outbreaks and epidemics on La Réunion, Mauritius, Mayotte, and the Seychelles (*6*), which caused considerable illness and death (*7*). Chikungunya appears to occur as an epidemic and an endemic disease in this region. The endemic disease affects mainly populations with high levels of IgG against CHIKV who live in rural areas in Africa (*8*). The epidemic disease occurs in Asia and the Indian Ocean region in populations in which herd immunity is weak, often in urban areas where *Aedes aegypti* and *Ae. albopictus* mosquitoes are the main transmission vectors. During epidemics, humans are the primary reservoirs. Monkeys, rodents, birds, and cattle have been identified as animal reservoirs (*9*–*11*). The onset of chikungunya epidemics is acute with high attack rates as seen in 2005–2006 on La Réunion (*12*). Concurrent epidemics of dengue and chikungunya have been reported from Asia (*13*) and Africa (*14*). In 2006, a combined outbreak of dengue fever and chikungunya fever occurred near the Madagascan city of Toamasina (*15*), but the rest of the country remained unaffected by this epidemic. An increased number of RVFV infections was noticed in Madagascar during the rainy seasons of 2008 and 2009 with 476 (19 fatal) and 236 (7 fatal) suspected cases, respectively (*4*).

The sudden emergence of chikungunya in the Mananjary area indicated a lack of herd immunity in the affected population. The previous outbreak of CHIKV infection in Madagascar in 2006 in the Toamasina region occurred in conjunction with DENV infection. Because of the recent reports of RVFV infections in animals and humans, our investigation of the recent outbreak in Madagascar included assessment of serologic parameters against CHIKV, DENV, and RVFV.

Approximately 2–3 months after the peak and 1–2 months after the decline of the outbreak of chikungunya, we retrospectively assessed the serologic markers and reported clinical features of women who came for routine pregnancy follow-up visits at 6 geographic locations. By focusing on pregnant women, we could reduce the need to stratify for age and sex and thus minimize the fragmentation of data. The focus could then to be placed on 1) assessing a possible inward spread of the epidemic, 2) evaluating whether the epidemic was limited to CHIKV or due to a simultaneous occurrence of DENV or RVFV infections, and 3) detecting factors associated with an increased risk of CHIKV infection.

## Methods

The cooperative project of the University of Antananarivo and the Bernhard Nocht Institute for Tropical Medicine was carried out during May–July 2010. Overall, 1,244 pregnant women were included from 6 different sites (Figure 1). Investigations were conducted in 2 coastal cities: Mananjary, the suspected epicenter of the chikungunya epidemic in February, and Manakara. Inland study sites included Ifanadiana, located at the ascending road from the above-mentioned cities to the highlands, the 2 highland cities of Tsiroanomandidy and Ambositra, and Moramanga, a highland city between Antananarivo and Toamasina. The study sites were chosen to include the coastal cities where the suspected chikungunya outbreak was reported (Manajary and Manakara), a city lying at the main road leading to the outbreak region on a moderate elevation level of 466 m (Ifanadiana), and 3 arbitrarily chosen cities in the highlands (Moramanga, Tsiroanomandidy, and Ambositra). Pregnancy follow-up services were chosen for a population comprehensive enough to allow the collection of ≈200 samples within a week. All women attending the routine pregnancy follow-up services were included.

The study was approved by the “Comité d’ethique de la Vice Primature Chargée de la Santé Publique” and discussed with representatives of the World Health Organization (WHO) during a meeting at the WHO office in Antananarivo on April 23, 2010. The study was explained to every participant, and informed consent was either signed (or, in the case of illiteracy, a fingerprint was obtained), and signature of a witness was acquired. Data on the course of pregnancy were obtained from interviews and review of the pregnancy follow-up booklet. To assess symptoms of CHIKV infections, researchers questioned the participants regarding current symptoms, symptoms that occurred since the time of their last menorrhea, or symptoms they recalled from a recent confirmed or suspected CHIKV infection.

**Figure 2:**
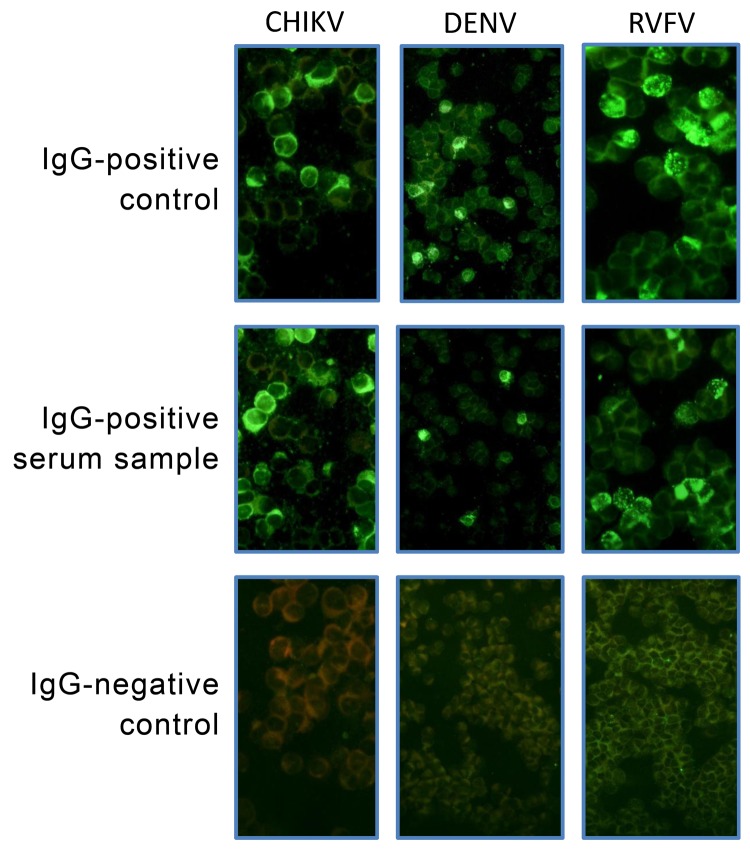
Images from immunofluorescence assays in Vero E6 cells for IgG against chikungunya virus (CHIKV), dengue virus (DENV), and Rift Valley fever virus (RVFV). Original magnification ×100 and ×200.

**Figure 3:**
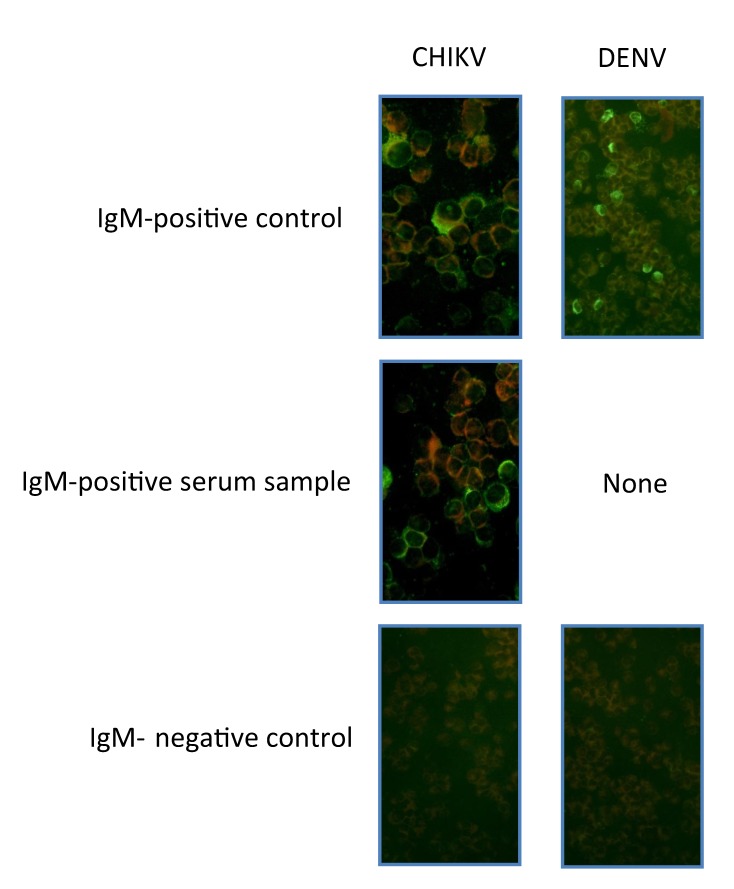
Images of immunofluorescence assays in Vero E6 cells for IgM against chikungunya virus (CHIKV) and dengue virus (DENV). For each of the viruses, a positive control, an example of a positive serum sample (if available), and a negative control are shown. Original magnification ×100 and × 200.

A venous blood sample collected into EDTA was taken for measurement of IgG against CHIKV, DENV, and RVFV. To detect acute CHIKV or DENV infections, we measured levels of IgM against CHIKV and DENV. Immunofluorescence assays (IFAs) for CHIKV, DENV, and RVFV were performed with virus-infected Vero E6 cells as described (*16*). In brief, Vero cells were spread onto slides, air dried, and fixed in acetone. Plasma samples were serially diluted in phosphate-buffered saline, starting with an initial dilution of 1:10, added to the cells, and incubated for 90 minutes at 37°C. After slides were washed with phosphate-buffered saline, they were incubated with fluorescein isothiocyanate–labeled rabbit antihuman IgG and IgM (SIFIN, Berlin, Germany) at 37°C for 25 minutes (Figure 2, Figure 3). IgG or IgM titers >100 were considered positive.

The serologic controls used for the IFA were the standard routine controls of the WHO Collaborating Centre for Arbovirus and Haemorrhagic Fever Reference and Research in Hamburg, Germany. Moreover, the controls used in this serologic survey were used as external quality assessment samples by the WHO Collaborating Centre for Quality Assurance and Standardization in Laboratory Medicine, Berlin, Germany. The sensitivity and specificity of the IFAs were demonstrated to be 100%, according to results of external quality assessments organized by the European Network for Diagnostics of Imported Viral Diseases (*17*). The DENV nonstructural protein 1 (NS1) antigen ELISA was performed according to the instructions of the manufacturer (Bio-Rad Laboratories, Hercules, CA, USA). The DENV NS1 antigen is a marker for DENV antigen circulation and thus detects acute infections up to day 21 after onset of symptoms.

The following retrospectively self-reported symptoms were assessed: fever, joint pain, and stiffness, skin symptoms and rashes, hip vibrations and pain, conjunctivitis, and stooped posture. For the 2 coastal locations where seroprevalence of CHIKV was highest, we analyzed potential risk factors for CHIKV infection, taking into account patient’s age, mosquito protective measures (bednet, fan, repellent, air conditioner), and because our study population consisted of pregnant women, we also considered pregnancy-related factors such as parity, weight, and trimester of pregnancy.

Statistical analysis was performed by using STATA version 10 (www.stata.com). The risk factor analysis was done by using univariable and multivariable logistic regressions. If the odds ratios suggested a trend over several categories, a nonparametric test for trend was conducted in the univariable analysis. For multivariable models, a Mantel-Haenszel test for trend, adjusted for the other variables in the model, was performed.

## Results

A total of 1,244 women from 6 locations in Madagascar were included in the study. All locations, when data were presented graphically, showed a right-skewed age distribution. The median age was 25 years (range 12–50 years). The median age of women from the 2 coastal locations (Mananjary and Manakara) was not significantly different from that of women from higher altitudes (p = 0.95, by Wilcoxon rank sum test). No relevant difference was found regarding the proportion of primiparas by location (28.4% in the highlands compared with 27.4% at the coast, p = 0.70, by χ^2^ test). None of the participants had an air conditioner at home; 3 women who lived on the coast reported using a fan. Two women who lived in the highlands reported using mosquito repellents. The only noteworthy mosquito protective measure used frequently was bednets (70.3%). Bednet use was significantly higher for women from the coastal cities of Mananjary (88.2%) and Manakara (90.8%) than for those living in the highland cities of Moramanga (65.7%), Ambositra (21.5%), and Tsiroanomandidy (56.7%). Of those from Ifanadiana, a coastal city at 450-m altitude, 94% used bednets.

Table 1 shows the levels of IgG against CHIKV, DENV, and RVFV, and the results of the IFAs that measured IgM against CHIKV and DENV. IgM against CHIKV was only detected in samples from Mananjary and Manakara taken ≈2–4 months after the peak of the epidemic. Although 27.5% of the samples from Mananjary and 5.2% of the samples from Manakara were positive for IgM against CHIKV, IgM against CHIKV was not detected in samples from other noncoastal locations. IgM against DENV and DENV NS1 antigen, indicators of DENV viremia, were not detected in any location.

**Table 1:** Measurement of antibodies against CHIKV, DENV, and RVFV in samples from pregnant women in Madagascar, May–July 2010*

Location	Altitude, m	Month	Total no. women	No. (%) women with				
				IgG against CHIKV	IgG against DENV	IgG against RVFV	IgG against CHIKV/DENV†	IgM against CHIKV‡
Mananjary	0 (coastal)	May	195	87 (44.6)	34 (17.4)	2 (1)	14 (7.2)	53 (27.5)
Manakara	0 (coastal)	Jun	251	57 (22.7)	27(10.8)	1 (0.4)	6 (2.4)	13 (5.2)
Ifanadiana	466	Jun	197	2 (1.0)	4(2.0)	0	0	0
Moramanga	920	Jul	198	6 (3.1)	23(11.7)	0	1 (0.5)	0
Tsiroanomandidy	860	Jul	203	0	8 (3.9)	1 (0.5)	0	0
Ambositra	1,280	May	175‡	2 (1.1)	1(0.6)	0	1 (0.6)	0

*CHIKV, chikungunya virus; DENV, dengue virus; RVFV, Rift Valley fever virus. †Samples that were positive for IgG against both CHIKV and DENV. ‡For 25 women from Ambositra, no serologic samples were taken. No samples were positive for IgM against DENV.

A total of 154 of the 1,244 pregnant women (12.4%) were positive for IgG against CHIKV, and 116 of them (75.3%) had reported a history of symptoms of CHIKV infection since their last menorrhea. The highest rates of IgG against CHIKV (Mananjary 44.6%, Manakara 22.7%) and IgM against DENV (Mananjary 17.4%, Manakara 10.8%) were found in the 2 coastal cities (Figure 1; Table 1). IgG against CHIKV and DENV were found in 7.2% and 2.4% in Mananjary and Manakara, respectively. Differences in seroprevalences at the other locations, all at higher altitudes, were negligible, apart from the high frequency of IgG against DENV in Moramanga (11.7%) and Tsiroanomandidy (3.9%). Among persons with IgG against CHIKV, the proportion of women who reported a history of symptoms related to CHIKV infection during the recent outbreak was the same in Mananjary and Manakara (79%). None of the women interviewed from Moramanga reported a history of recent symptoms, even if they had IgG against CHIKV or DENV.

According to the results, only persons with IgG against CHIKV from Mananjary and Manakara could be confidently assigned to the 2009–2010 outbreak. Therefore, reported symptoms and analysis of risk factors for CHIKV infection was confined to these 2 locations (Table 2, Table 3). Risk for previous CHIKV infection increased with body weight (Table 2). This association persisted after adjusting for parity, bednet use, and age (Table 3) and after additionally adjusting for the trimester of pregnancy (Table 3).

**Table 2:** Signs and symptoms of pregnant women with IgG against CHIKV from Mananjary and Manakara, Madagascar, May–July 2010*

Symptom/sign	No. (%) women with	
	IgG against CHIKV, n = 144	IgG against DENV, n = 61
Fever	107 (74)	21 (34)
Joint pain and stiffness	113 (78)	22 (36)
Skin symptoms or rashes	87 (60)	19 (31)
Hip vibrations and pain	84 (58)	20 (33)
Stooped posture†	77 (53)	18 (30)
Conjunctivitis	27 (18)	9 (15)
Asymptomatic	30 (21)	39 (64)

*CHIKV, chikungunya virus; DENV, dengue virus. †Stooped posture refers to the posture that persons affected by strong joint and muscle pains adopt to relieve pain. The word “chikungunya” comes from the Makonde language spoken in southeast Tanzania and means “the one who bends up and curls like a drying leaf.”

**Table 3:** Protective or risk factors for CHIKV infection in pregnant women from 2 coastal locations Mananjary (N = 195) and Manakara (N = 251), Madagascar, May–July 2010*

Factor	No. women	Bivariable analysis, n = 446			Multivariable model 1, n = 433			Multivariable model 2, n = 376	
		OR (95% CI)†	p value		OR (95% CI)	p value		OR (95% CI)	p value
Bednet use									
No	40	Reference	0.70		Reference	0.98		Reference	0.95
Yes	400	0.87 (0.44–1.73)			0.99 (0.47–2.06)			1.02 (0.47–2.22)	
Parity									
Multipara	324	Reference	0.93		Reference	0.44		Reference	0.65
Primipara	122	0.98 (0.63–1.53)			0.79 (0.43–1.44)			0.86 (0.44–1.67)	
Age, y									
<20	94	Reference	0.67†		Reference	0.17		Reference	0.37
20–30	225	0.82 (0.49–1.36)			0.70 (0.36–1.34)			0.81 (0.39–1.69)	
>30	125	0.87 (0.49–1.53)			0.57 (0.26–1.23)			0.68 (0.29–1.60)	
Weight, kg									
<40	17	Reference	0.001†		Reference	0.0006		Reference	0.001
40–49	242	1.22 (0.38–3.87)			1.22 (0.38–3.92)			1.45 (0.39–5.45)	
50–59	142	1.82 (0.56–5.88)			1.86 (0.57–6.08)			2.36 (0.61–9.09)	
60–70	32	2.53 (0.67–9.47)			2.84 0.74–10.8			3.82 (0.85–17.2)	
>70	8	9.75 (1.38–68.8)			10.8 (1.52–77.2)			11.4 (1.39–93.0)	
Trimester									
1st	9	Reference	0.45					Reference	0.39‡
2nd	142	0.37 (0.10–1.45)						0.44 (0.10–1.90)	
3rd	236	0.36 (0.10–1.40)						0.40 (0.10–1.71)	

*CHIKV, chikungunya virus; OR, odds ratio. †Nonparametric test of trend. ‡Mantel-Haenszel test for trend, adjusted for the other variables.

## Discussion

In contrast to the outbreak in the Toamasina area of Madagascar in 2006, the outbreak investigated here appears to be exclusively caused by CHIKV infections without concomitant DENV infection. Although one third of the participants in coastal cities at the epicenter of the outbreak were infected with CHIKV, the epidemic did not spread to higher altitudes and further inland. The chikungunya epidemic curve reached its peak in Mananjary in February and abated in March 2010; in Manakara, the epidemic occurred ≈1 month later. The duration of the epidemic roughly corresponds to the rainy season in Madagascar from November to April. Anti-CHIKV IgG was detected in all samples positive for anti-CHIKV IgM, and we assumed that the outbreak had ended before the investigation described here was started. In the noncoastal locations, all samples were negative for IgM against CHIKV, which suggests that the epidemic was constrained to the coast.

By focusing on pregnant women, we achieved a relatively homogenous study population, which facilitated the comparison of differences between study sites by reducing differences between persons (e.g., age or sex) and overfragmentation of the data. In addition to DENV, the presence of RVFV was assessed because this virus has been recently reported in Madagascar (*4*,*18*). In 22 persons, IgG against CHIKV and IgG against DENV were detected. The major mosquito vectors of both viruses are equally susceptible to CHIKV and DENV, with simultaneous transmission being confirmed in experimental settings (*19*,*20*). Although a considerable number of the samples from Mananjary (17.4%) and from Manakara (10.8%) were positive for IgG against DENV, none was positive for DENV IgM or DENV NS1 antigen, which suggests past DENV infections, independent of the recent chikungunya epidemic.

A limitation of the study is the possibility that some women may have had prior CHIKV infections before the outbreak investigated in this study. A CHIKV outbreak was reported in the Toamasina region in 2006 (*15*). However, it seems unlikely that a relevant proportion of the resident population of the Mananjary region acquired CHIKV immunity during the Toamasina outbreak because this region is 400 km distant from Mananjary, and no tarred road exists along the coast. The elevated seroprevalance of IgG and IgM against CHIKV in the coastal city of Manakara, which lies 100 km south of Mananjary, suggests that the outbreak spread southwards along the coast.

The International Federation of Red Cross and Red Crescent Societies reported that the town of Irondo, where the inbound roads from Mananjary and Manakara meet, was also affected by the outbreak (*2*).The town is situated 30 km from the coastline at an altitude of ≈40 m. Although an altitude of 400–500 m does not avert mosquito survival, the low seroprevalence in Ifanadiana (altitude 466 m, 70 km inland) provides evidence against an upward and inbound spread of the epidemic. The long travel distance between Irondro and Ifanadiana, with serpentine roads and long uninhabited stretches between small settlements, may have interrupted transmission chains. Samples taken from the highland cities of Ambositra (1,280 m), Tsiroanomandidy (860 m), and Moramanga (920 m) indicate that the highland population of Madagascar remained unaffected by the recent chikungunya outbreak.

According to our data, 21% of the women from the coastal cities who were positive for IgG against CHIKV did not report symptoms corresponding to those of chikungunya during the outbreak. Similarly, the proportion of asymptomatic infections during a CHIKV outbreak in a naïve population from northeastern Italy in 2007 was 18% (*21*). In contrast, during the 2005–2006 chikungunya outbreak on La Réunion, only 5% of infections remained asymptomatic (*22*,*23*).

Entomologic data were not collected during the chikungunya outbreak on Madagascar. The main vectors for CHIKV are *Ae. albopictus* and *Ae. aegypti* mosquitoes*.* In a study from Gabon, *Ae. albopictus* mosquitoes outnumbered *Ae. aegypti* mosquitoes in most suburban areas, and in urban areas, where *Ae. aegypti* mosquitoes were more commonly found, CHIKV and DENV were only found in *Ae. albopictus* (*19*) mosquitoes. *Ae. albopictus* mosquitoes were the main vectors on La Réunion during the 2005–2006 epidemic.

Recent entomologic data for Madagascar were scarce in 2010, when the chikungunya outbreak took place. In a report from 1989, *Ae. albopictus* was identified as the predominant *Aedes* species on the eastern coast and on the central highland plateau, where all our study sites were located (*24*). A recent study provides evidence for an expansion of an invasive lineage of *Ae. albopictus* that has spread throughout the island and possibly caused a decline of *Ae. aegypti* at least in urban areas (*25*). More research on the vector dynamics of *Ae. albopictus* and *Ae. aegypti* mosquitoes in Madagascar is needed.

The analysis of risk and protective factors for infection was confined to the epidemic coastal cities. The finding that bednet use had no influence on the risk of CHIKV infection is in accordance with the fact that the chikungunya vectors *Ae. albopictus* and *Ae. aegypti* mosquitoes bite during the daytime. The results indicate a positive association between body weight and the risk for CHIKV infection but the causality could not be assessed in this cross-sectional study. Notably, a study from India found evidence of a higher risk for chronic sequelae among obese persons who acquired chikungunya (*26*). Future studies on risk factors for chikungunya should include body weight as a possible influence.

The 2009–2010 arboviral outbreak in coastal eastern Madagascar was an isolated CHIKV epidemic without relevant DENV co-transmission. With more than one third of all women affected in the epicenter, the infection rate in the population was high. Data from other locations suggest that the epidemic did not spread to higher altitudes and inland.
